# Association between changes in obesity status and neuropsychiatric health and brain structure in different glucose status

**DOI:** 10.3389/fnut.2025.1676168

**Published:** 2025-10-03

**Authors:** Ping Chen, Pan Zhang, Zhiwei Lin, Jianmin Qiu, Min Zou, Ruilin Liu, Lifang Huang, Wen Sun

**Affiliations:** ^1^Department of Neurology, The First Hospital of Putian City, Putian, China; ^2^The Graduate School of Fujian Medical University, Fuzhou, China; ^3^Department of Neurology, Centre for Leading Medicine and Advanced Technologies of IHM, The First Affiliated Hospital of USTC, Division of Life Sciences and Medicine, University of Science and Technology of China, Hefei, China

**Keywords:** obesity, neuropsychiatric disorders, brain structure, glycemic status, UK biobank

## Abstract

**Background:**

Obesity is a major global health challenge, linked to cardiometabolic and neuropsychiatric disorders through mechanisms such as inflammation and insulin resistance. However, little is known about how adiposity and its longitudinal changes interact with glycemic status to shape neuropsychiatric health and brain structural vulnerability. Clarifying these relationships is of high importance, as both obesity and dysglycemia are modifiable risk factors that may jointly accelerate psychiatric disorder and brain aging.

**Methods:**

Using UK Biobank data (*n* = 423,750, with 32,551 having brain MRI), we examined associations between obesity indicators (body mass index [BMI], waist circumference [WC], body fat percentage [BFP]) and changes in obesity status with incident neuropsychiatric disorders (stroke, dementia, Parkinson’s disease, depression, anxiety) and brain structural measures. Participants were stratified by glycemic status—normal glucose regulation (NGR), prediabetes (Pre-DM), and diabetes (DM)—based on American Diabetes Association criteria. Cox proportional hazards and linear regression models were used.

**Results:**

Higher BMI, WC, and BFP were associated with increased risks of depression and anxiety across all glycemic groups, particularly in NGR. Abdominal obesity was linked to Parkinson’s disease risk in NGR. Conversely, BMI showed an inverse association with dementia in NGR, possibly due to reverse causality. Persistent obesity and weight gain were associated with higher depression and anxiety risks in NGR. In diabetes, higher BFP was strongly linked to reduced grey matter, thalamus, and hippocampus volumes and increased WMHs. This association with BFP represented the most robust imaging signal, highlighting the pronounced vulnerability of brain structure to excess adiposity in diabetes. Similar but weaker patterns were observed in prediabetes and NGR.

**Conclusion:**

Obesity, particularly persistent or increasing adiposity, adversely affects neuropsychiatric health and brain structure, and these effects are significantly modified by glycemic status. Our findings underscore the importance of considering glucose metabolism when assessing obesity-related brain risks, and suggest that early weight management and metabolic control may have broad benefits for preventing neuropsychiatric disorders and mitigating brain aging.

## Introduction

Obesity has emerged as one of the most significant global public health challenges, with its prevalence rising steadily over recent decades (1). It is well established that obesity contributes not only to cardiometabolic conditions—such as type 2 diabetes, hypertension, and cardiovascular disease—but also to adverse mental health outcomes and neurological disorders (2, 3). Increasing evidence suggests that excess adiposity may elevate the risk of neuropsychiatric conditions possibly through systemic inflammation, insulin resistance, vascular dysfunction, and neuroendocrine disturbances (4, 5).

Notably, obesity is a heterogeneous condition, varying in distribution (e.g., general vs. abdominal adiposity) and composition (e.g., fat vs. lean mass). Different indicators of obesity—such as body mass index (BMI), waist circumference, and body fat percentage—may capture distinct aspects of body composition and confer differential health risks (6). However, the extent to which these diverse obesity phenotypes relate to neuropsychiatric and brain structural outcomes remains incompletely understood.

Moreover, metabolic health—particularly glycemic status—may significantly modify the impact of obesity on the brain. Individuals with impaired glucose regulation, such as those with prediabetes or diabetes, often exhibit higher levels of systemic inflammation, oxidative stress, and vascular injury, all of which are implicated in brain aging and psychiatric vulnerability (7, 8). Despite this, few large-scale studies have systematically explored how the relationship between obesity and neuropsychiatric or neurostructural outcomes varies across the glycemic spectrum. To address this gap, participants in the present study were stratified by glycemic status according to the American Diabetes Association: normal glucose regulation (NGR), prediabetes (Pre-DM), and diabetes (DM).

Emerging evidence also suggests that glycemic status itself may causally shape neuropsychiatric vulnerability and brain outcomes (9). Chronic hyperglycemia and insulin resistance, hallmarks of prediabetes and diabetes, can impair neuronal glucose utilization, promote oxidative stress, and disrupt synaptic plasticity, thereby accelerating neurodegeneration (10). These disturbances often manifest in structural brain alterations such as reduced grey matter, hippocampal, and thalamic volumes, as well as increased white matter hyperintensities—markers of cerebral small vessel disease and cognitive decline (11). In addition, hyperglycemia-driven systemic inflammation and pro-inflammatory cytokine release (e.g., IL-6, TNF-*α*) may cross the blood–brain barrier, contributing to neuroinflammation and mood dysregulation (12). Dysregulation of the hypothalamic–pituitary–adrenal (HPA) axis and reduced neurotrophic factors such as brain-derived neurotrophic factor (BDNF) further link impaired glucose metabolism with depression, anxiety, and memory impairment (13). Importantly, these mechanisms may operate in a graded fashion, with subtle effects present in prediabetes and more pronounced changes in diabetes, while individuals with normal glucose regulation may display different or paradoxical associations (e.g., reverse causality between BMI and dementia) (14). Given this biological rationale, stratifying analyses by glycemic status provides a critical opportunity to disentangle how obesity interacts with metabolic health to influence neuropsychiatric disorders and brain structural vulnerability.

Beyond static obesity status, dynamic changes in body composition may offer additional prognostic insight. Weight gain, persistent obesity, and even weight loss may reflect underlying health trajectories that are differentially associated with mental and cognitive outcomes. Investigating these longitudinal changes is essential for understanding the temporal nature of obesity-related neurobiological consequences and for informing targeted interventions.

Despite increasing recognition of the adverse impact of obesity on brain and mental health, most prior studies have relied on static measurements of adiposity, focusing on single time points rather than longitudinal trajectories. This approach does not capture the dynamic nature of obesity, where weight gain, weight loss, or persistent obesity may reflect distinct health trajectories with potentially different neuropsychiatric consequences. Moreover, limited evidence exists on how these dynamic changes interact with glycemic status to influence neuropsychiatric outcomes and brain structural alterations. Addressing this gap is critical, as both obesity and impaired glucose regulation are modifiable risk factors, and understanding their joint impact may provide novel insights into prevention strategies.

Based on this rationale, we hypothesized that: (1) higher adiposity and sustained or increasing obesity would be associated with elevated risks of neuropsychiatric disorders and adverse brain structural changes, whereas weight loss might also indicate unfavorable outcomes due to underlying health conditions; and (2) these associations would be more pronounced among individuals with impaired glycemic status (prediabetes and diabetes) compared to those with normal glucose regulation.

To address these gaps, we conducted a comprehensive analysis using data from the UK Biobank, a large, prospective, population-based cohort with extensive phenotypic and neuroimaging data. Our study aimed to (1) examine the associations between multiple obesity indicators and the incidence of neuropsychiatric disorders; (2) explore how changes in obesity status relate to neuropsychiatric and structural brain outcomes; and (3) investigate whether these associations differ according to baseline glycemic status. By integrating anthropometric, clinical, and imaging data, our findings provide novel insights into the interplay between metabolic health, body composition, and brain health.

## Method

### Study population

The UK Biobank^1^ is a large, population-based cohort comprising 503,325 individuals aged 45–69 years, recruited across the United Kingdom over a five-year period beginning in 2006 (15). The study was approved by the National Research Ethics Service Committee North West–Haydock (reference 11/NW/0382), and all participants provided informed consent. All procedures adhered to the ethical principles outlined in the Declaration of Helsinki. For the present analysis, after excluding individuals with baseline neuropsychiatric disorders (*n* = 69,789) and missing obesity-related measurements (*n* = 8,730), a total of 423,750 participants were included, of whom 32,551 had available brain structural imaging data.

### Obesity indicators

Obesity indicators included BMI, waist circumference, and body fat percentage. Based on these, we defined three obesity types: general obesity, abdominal obesity, and high body fat percentage. BMI was calculated as weight in kilograms divided by height in meters squared (kg/m^2^). General obesity was defined as BMI ≥ 30 kg/m^2^ (16); abdominal obesity as waist circumference ≥ 102 cm for men or ≥ 88 cm for women (17); and high body fat percentage as ≥ 25% for men or ≥ 35% for women (18). Changes in these obesity types were categorized as: remained normal, increased, decreased, or remained obesity (general, abdominal, or high body fat).

### Neuropsychiatric disorders outcomes

Outcomes were identified through linkage with hospital inpatient records from the Hospital Episode Statistics (HES) in England, the Scottish Morbidity Record, and the Patient Episode Database for Wales. These sources provided detailed data on hospital admissions and diagnoses, coded using the International Classification of Diseases, 10th Revision (ICD-10). The primary outcomes included incident stroke (I60–I64), dementia (F00–F05, G30–G31), Parkinson’s disease (G20), depressive disorder (F32–F33), and anxiety disorder (F40–F48).

### Brain volume measurement

Brain structure data were obtained from magnetic resonance imaging (MRI) starting in 2014.^2^ Volumes of the whole brain, white matter, grey matter, thalamus, and hippocampus were derived from T1-weighted images, while WMHs were obtained from T2-weighted scans. Brain volumes were normalized for head size using estimates of skull surface from T1 images, summed across hemispheres, and then z-standardized. WMHs volume was log-transformed prior to z-standardization due to skewness. Neurodegeneration-related brain volumes—including total brain, white matter, WMHs, grey matter, thalamus, and hippocampus—served as continuous outcomes in this study.

### Definitions of glucose metabolism status

Based on the criteria of the American Diabetes Association (ADA), participants were classified into three glycemic status groups: normal glucose regulation (NGR; fasting blood glucose [FBG] < 5.6 mmol/L and HbA1c < 5.7%, with no use of glucose-lowering medications), prediabetes (Pre-DM; FBG 5.6–6.9 mmol/L or HbA1c 5.7–6.4%, without medication use), and diabetes (DM; FBG ≥ 7.0 mmol/L, HbA1c ≥ 6.5%, or current use of glucose-lowering medications) (19).

### Assessment of covariates

Baseline characteristics were obtained through demographic data, lifestyle assessments, medical history, and physical examinations. Demographic variables included age, sex, and ethnicity. Lifestyle factors—such as smoking status and alcohol consumption—and medical histories of hypertension and hypercholesterolemia were collected through questionnaires, interviews, and linked medical records. Socioeconomic status was assessed using the Townsend Deprivation Index (TDI), a measure derived from national census data that reflects material deprivation (20). Physical activity was measured using the International Physical Activity Questionnaire, and metabolic equivalent of task (MET) scores were calculated accordingly. Sedentary behavior was defined as time spent driving, watching television, or using a computer.

### Statistical analysis

Categorical variables were summarized as frequencies and percentages, skewed continuous variables as medians with interquartile ranges (IQR), and normally distributed continuous variables as means ± standard deviations (SD). To estimate hazard ratios (HRs) and 95% confidence intervals (CIs) for the associations between obesity indicators—including changes in these indicators—and the incidence of neuropsychiatric disorders across different glycemic statuses, Cox proportional hazards models were employed. These models were adjusted for age, sex, ethnicity, TDI, smoking status, alcohol consumption, hypertension, hypercholesterolemia, sleep duration, physical inactivity, and sedentary behavior. Linear regression models were used to calculate *β* coefficients and 95% CIs for the associations between adiposity indicators, their changes, and brain structural measures among participants with available neuroimaging data, stratified by glycemic status. For continuous obesity indicators (BMI, WC, BFP), HRs were estimated per 1-unit increase. For categorical definitions of obesity (general, abdominal, high body fat) and for changes in obesity status (e.g., persistent, incident, reversed), HRs were calculated with the normal group as the reference. To control for type I error due to multiple testing, we applied false discovery rate (FDR) correction using the Benjamini–Hochberg method across all analyses.

All statistical analyses were conducted using R software (version 4.3.1). Two-sided *p*-values were reported, with statistical significance set at *p* < 0.05.

## Results

### Baseline characteristics

The flow chart is shown in Supplementary Figure S1. A total of 423,750 participants were included in the study. Among them, 11,975 (2.8%) were diagnosed with stroke, 8,478 (2.0%) with dementia, 2,940 (0.7%) with Parkinson’s disease, 18,037 (4.3%) with depression, and 21,631 (5.1%) with anxiety. The median age of the overall cohort was 58 years (IQR: 50–63). Participants with neurological or psychiatric conditions were generally older, with median ages ranging from 57 years in the depression group to 65 years in the dementia group. Individuals with neurological or psychiatric conditions had higher proportions of prediabetes and diabetes compared to the overall population. 15.3% of stroke patients had prediabetes and 7.9% had diabetes, compared to 11.9 and 3.9%, respectively, in the total sample (Supplementary Table S1).

### Obesity indicators and neuropsychiatric health in different glucose metabolic states

**Figure 1:**
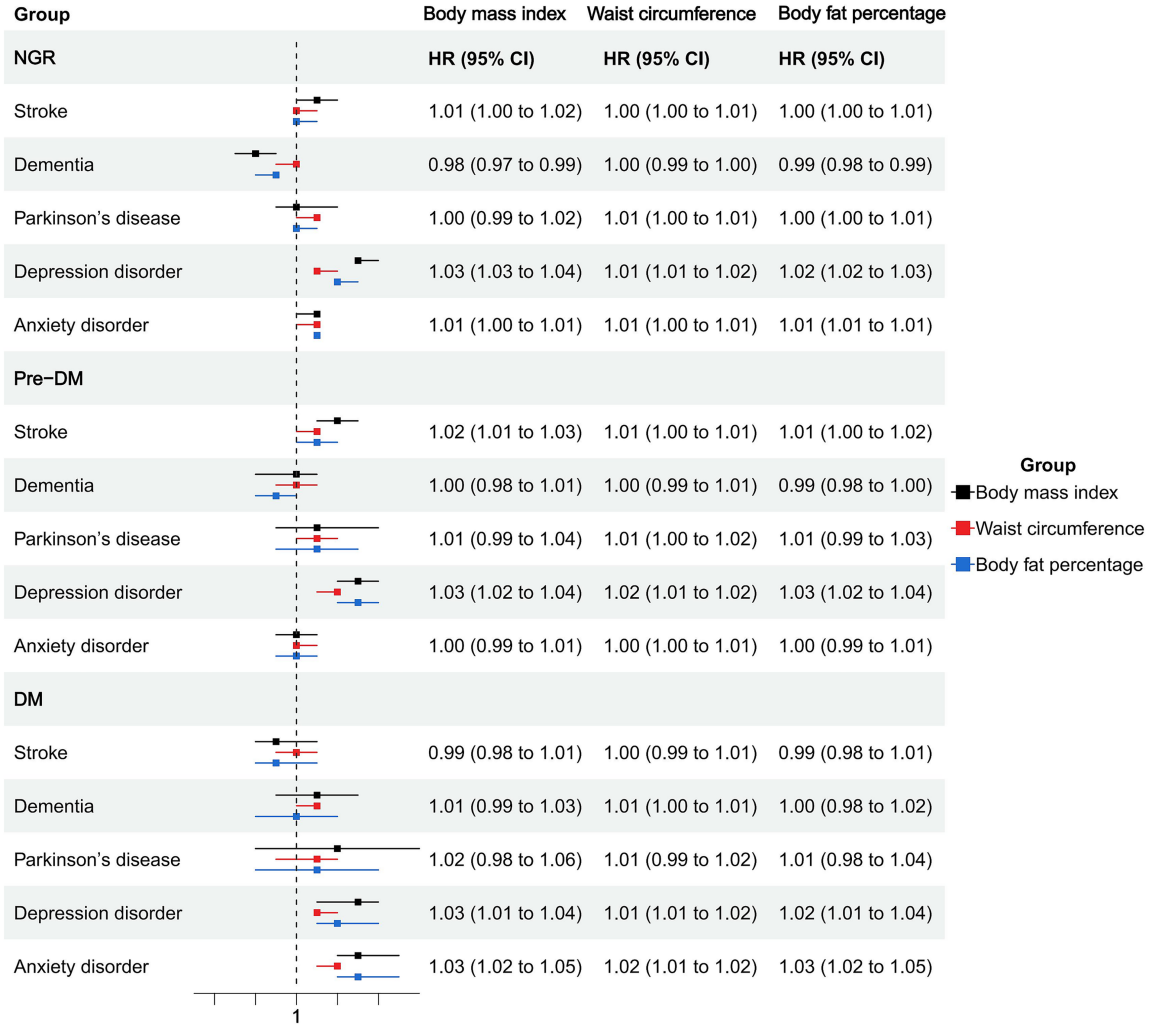
Association of obesity indicators and neuropsychiatric health in different glucose metabolic states.

**Figure 2:**
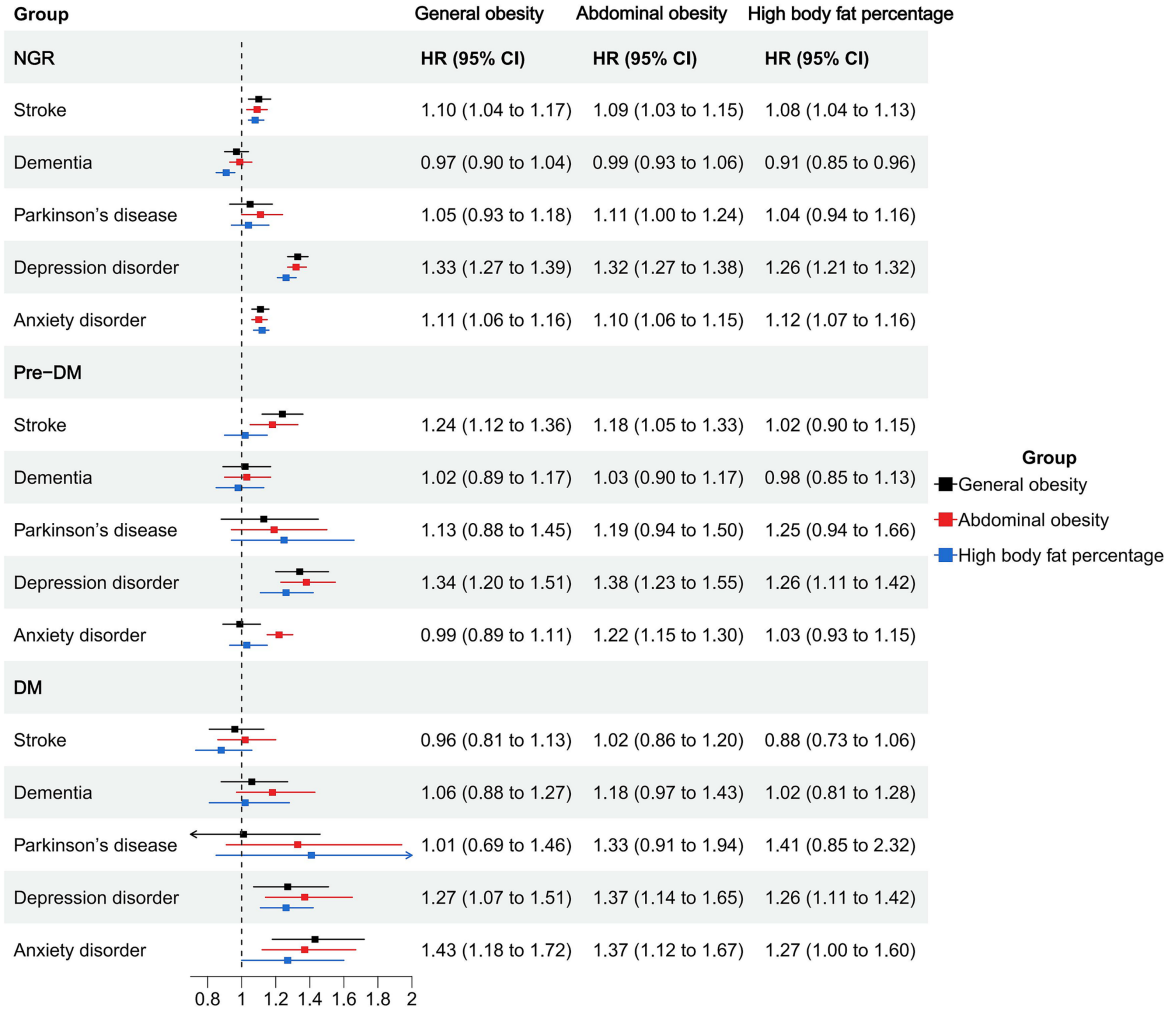
Association of obesity types and neuropsychiatric health in different glucose metabolic states.

Among individuals with NGR, higher BMI was associated with an increased risk of stroke (HR = 1.01, 95% CI: 1.00–1.02), depression (HR = 1.03, 95% CI: 1.03–1.04), and anxiety (HR = 1.01, 95% CI: 1.00–1.01), whereas it was inversely associated with dementia (HR = 0.98, 95% CI: 0.97–0.99). Waist circumference and body fat percentage showed similar trends, with significant positive associations for stroke, depression, and anxiety, and an inverse association between body fat percentage and dementia (HR = 0.99, 95% CI: 0.98–0.99). General, abdominal, and high body fat-defined obesity were consistently associated with elevated risks of depression (HRs ranging from 1.26 to 1.33) and anxiety (HRs ranging from 1.10 to 1.12). Notably, abdominal obesity was associated with an increased risk of Parkinson’s disease (HR = 1.11, 95% CI: 1.00–1.24; Figures 1, 2; Supplementary Table S2).

In the prediabetic group, BMI and waist circumference remained significantly associated with elevated depression risk (HR = 1.03, 95% CI: 1.02–1.04 and HR = 1.02, 95% CI: 1.01–1.02). General and abdominal obesity were also robustly associated with depression (HR = 1.34, 95% CI: 1.20–1.51 and HR = 1.38, 95% CI: 1.23–1.55), and abdominal obesity was associated with anxiety (HR = 1.22, 95% CI: 1.15–1.30). High body fat percentage showed a significant association with depression (HR = 1.26, 95% CI: 1.11–1.42) but not with anxiety (Figures 1, 2; Supplementary Table S2).

Among participants with diabetes, most obesity indicators were not significantly associated with stroke, dementia, or Parkinson’s disease. However, BMI, waist circumference, and body fat percentage were all significantly associated with increased risks of depression and anxiety. Specifically, BMI was associated with depression (HR = 1.03, 95% CI: 1.01–1.04) and anxiety (HR = 1.03, 95% CI: 1.02–1.05). Abdominal obesity showed strong associations with depression (HR = 1.37, 95% CI: 1.14–1.65) and anxiety (HR = 1.37, 95% CI: 1.12–1.67). General obesity was also positively associated with these two outcomes (depression: HR = 1.27; anxiety: HR = 1.43). High body fat percentage was significantly associated with depression (HR = 1.26, 95% CI: 1.11–1.42) and anxiety (HR = 1.27, 95% CI: 1.00–1.60; Figures 1, 2; Supplementary Table S2).

### Change in obesity status and neuropsychiatric health outcomes in different glucose metabolic states

The association of changes in weight with neuropsychiatric health was shown in Table 1. Among individuals with NGR, both weight gain and persistent obesity were significantly associated with increased risks of depression (HR = 1.57, 95% CI: 1.29–1.90 and HR = 1.29, 95% CI: 1.11–1.49, respectively) and anxiety (HR = 1.39, 95% CI: 1.17–1.66 and HR = 1.16, 95% CI: 1.02–1.32, respectively). Weight loss was also associated with a significantly increased risk of depression (HR = 1.41, 95% CI: 1.10–1.79), though its effect on anxiety was not statistically significant. For stroke, only persistent obesity showed a significant association (HR = 1.28, 95% CI: 1.05–1.56), while no significant associations were observed for dementia or Parkinson’s disease across all weight change groups in this subgroup.

**Table 1:** Association of changes in weight with neuropsychiatric health.

	Stroke			Dementia			Parkinson’s disease			Depression			Anxiety		
NGR	HR (95%CI)	P	FDR adjustment P	HR (95%CI)	P	FDR adjustment P	HR (95%CI)	P	FDR adjustment P	HR (95%CI)	P	FDR adjustment P	HR (95%CI)	P	FDR adjustment P
Maintained normal	1 (reference)			1 (reference)			1 (reference)			1 (reference)			1 (reference)		
Weight gain	1 (0.71–1.41)	0.987	0.987	0.71 (0.36–1.39)	0.319	0.634	0.56 (0.21–1.51)	0.25	0.783	1.57 (1.29–1.9)	<0.001	<0.001	1.39 (1.17–1.66)	<0.001	0.001
Weight loss	1.33 (0.96–1.83)	0.082	0.123	0.98 (0.55–1.76)	0.952	0.998	0.87 (0.38–1.98)	0.735	0.945	1.41 (1.1–1.79)	0.006	0.007	1.08 (0.85–1.37)	0.542	0.845
Persistent obesity	1.28 (1.05–1.56)	0.013	0.039	0.98 (0.69–1.39)	0.895	0.998	1.11 (0.72–1.72)	0.636	0.945	1.29 (1.11–1.49)	0.001	0.001	1.16 (1.02–1.32)	0.024	0.034
Pre-DM															
Maintained normal	1 (reference)			1 (reference)			1 (reference)			1 (reference)			1 (reference)		
Weight gain	1.19 (0.51–2.73)	0.689	0.689	1.76 (0.53–5.83)	0.352	0.634	0 (0 - Inf)	0.998	0.999	1.3 (0.67–2.5)	0.437	0.665	1.02 (0.55–1.9)	0.946	0.946
Weight loss	0.5 (0.18–1.37)	0.179	0.268	0.34 (0.05–2.52)	0.291	0.634	1.44 (0.32–6.59)	0.635	0.945	1.07 (0.56–2.07)	0.837	0.837	0.7 (0.36–1.38)	0.304	0.535
Persistent obesity	1.42 (0.93–2.17)	0.102	0.268	0.72 (0.29–1.79)	0.485	0.728	1.76 (0.66–4.74)	0.261	0.783	1.29 (0.89–1.88)	0.178	0.534	1.35 (0.99–1.85)	0.058	0.348
DM															
Maintained normal	1 (reference)			1 (reference)			1 (reference)			1 (reference)			1 (reference)		
Weight gain	3.14 (1.18–8.4)	0.022	0.066	0 (0 -Inf)	0.998	0.998	3.82 (0.39–37.33)	0.249	0.783	0.84 (0.2–3.55)	0.812	0.998	2.04 (0.59–7.03)	0.26	0.535
Weight loss	1.32 (0.52–3.4)	0.561	0.676	2.09 (0.53–8.21)	0.29	0.634	0 (0 -Inf)	0.999	0.999	0.78 (0.27–2.3)	0.658	0.823	0.5 (0.11–2.19)	0.357	0.535
Persistent obesity	0.85 (0.4–1.81)	0.676	0.676	1.97 (0.71–5.43)	0.19	0.634	1.96 (0.37–10.49)	0.431	0.945	1.26 (0.7–2.29)	0.444	0.740	1.11 (0.53–2.32)	0.787	0.944

The association of changes in abdominal obesity with neuropsychiatric health was shown in Table 2. In individuals with NGR, increased abdominal obesity was significantly associated with higher risks of depression (HR = 1.59, 95% CI: 1.38–1.84) and anxiety (HR = 1.21, 95% CI: 1.06–1.38), while reversed abdominal obesity also conferred elevated risks for depression (HR = 1.35, 95% CI: 1.09–1.67). Persistent abdominal obesity was linked to significantly increased risks of stroke (HR = 1.27, 95% CI: 1.06–1.52) and depression (HR = 1.36, 95% CI: 1.19–1.56), and showed a borderline association with anxiety (HR = 1.12, 95% CI: 0.99–1.26). No statistically significant associations were observed with dementia or Parkinson’s disease in this subgroup. Among prediabetic individuals, increased abdominal obesity was significantly associated with a higher risk of depression (HR = 1.65, 95% CI: 1.02–2.68). Reversed abdominal obesity was also significantly linked to an increased risk of depression (HR = 1.92, 95% CI: 1.13–3.24). In the DM group, no associations were observed between changes in abdominal obesity and any neuropsychiatric health outcomes.

**Table 2:** Association of changes in abdominal obesity with neuropsychiatric health.

	Stroke			Dementia			Parkinson’s disease			Depression			Anxiety		
NGR	HR (95%CI)	P	FDR adjustment P	HR (95%CI)	P	FDR adjustment P	HR (95%CI)	P	FDR adjustment P	HR (95%CI)	P	FDR adjustment P	HR (95%CI)	P	FDR adjustment P
Maintained normal WC	1 (reference)			1 (reference)			1 (reference)			1 (reference)			1 (reference)		
Increased abdominal obesity	1.23 (0.99–1.53)	0.064	0.086	0.73 (0.49–1.1)	0.131	0.393	0.99 (0.59–1.67)	0.983	0.999	1.59 (1.38–1.84)	<0.001	<0.001	1.21 (1.06–1.38)	0.005	0.045
Reversed abdominal obesity	1.27 (0.97–1.65)	0.086	0.086	0.74 (0.44–1.23)	0.246	0.443	1.15 (0.63–2.09)	0.656	0.881	1.35 (1.09–1.67)	0.007	0.017	0.99 (0.81–1.22)	0.944	0.944
Persistent abdominal obesity	1.27 (1.06–1.52)	0.01	0.03	0.91 (0.67–1.23)	0.523	0.672	1.11 (0.74–1.66)	0.626	0.913	1.36 (1.19–1.56)	<0.001	<0.001	1.12 (0.99–1.26)	0.067	0.184
Pre-DM															
Maintained normal WC	1 (reference)			1 (reference)			1 (reference)			1 (reference)			1 (reference)		
Increased abdominal obesity	0.97 (0.51–1.86)	0.937	0.937	0.66 (0.2–2.22)	0.504	0.672	1.75 (0.47–6.48)	0.403	0.999	1.65 (1.02–2.68)	0.042	0.076	1.22 (0.79–1.89)	0.369	0.664
Reversed abdominal obesity	0.76 (0.35–1.67)	0.497	0.786	1.06 (0.36–3.1)	0.919	0.919	1.12 (0.24–5.23)	0.881	0.881	1.92 (1.13–3.24)	0.015	0.034	1.52 (0.95–2.44)	0.082	0.184
Persistent abdominal obesity	1.35 (0.89–2.03)	0.156	0.597	0.56 (0.24–1.3)	0.175	0.394	1.06 (0.37–3.09)	0.913	0.913	1.43 (0.99–2.08)	0.059	0.088	1.32 (0.97–1.8)	0.081	0.184
DM															
Maintained normal WC	1 (reference)			1 (reference)			1 (reference)			1 (reference)			1 (reference)		
Increased abdominal obesity	1.78 (0.74–4.27)	0.199	0.597	3.99 (0.88–18.15)	0.073	0.328	0(0 - Inf)	0.999	0.999	1.36 (0.55–3.4)	0.506	0.545	0.64 (0.14–2.82)	0.553	0.789
Reversed abdominal obesity	1.31 (0.57–3.05)	0.524	0.786	0.81 (0.09–7.35)	0.851	0.919	1.3 (0.13–13.09)	0.824	0.881	0.7 (0.24–2.05)	0.51	0.545	0.77 (0.22–2.7)	0.682	0.789
Persistent abdominal obesity	0.91 (0.46–1.79)	0.784	0.937	3.3 (1–10.88)	0.05	0.328	1.47 (0.27–7.85)	0.654	0.913	1.21 (0.66–2.23)	0.545	0.545	1.16 (0.55–2.44)	0.701	0.789

The association of changes in body fat percentage with neuropsychiatric health was shown in Table 3. While changes in body fat percentage showed no significant association with most neuropsychiatric outcomes, increased (HR 1.36, 95% CI 1.14–1.63) and persistently high body fat (HR 1.32, 95% CI 1.15–1.51) were significantly associated with an elevated risk of depression in individuals with NGR.

**Table 3:** Association of changes in body fat percentage with neuropsychiatric health.

	Stroke			Dementia			Parkinson’s disease			Depression			Anxiety		
NGR	HR (95%CI)	P	FDR adjustment P	HR (95%CI)	P	FDR adjustment P	HR (95%CI)	P	FDR adjustment P	HR (95%CI)	P	FDR adjustment P	HR (95%CI)	P	FDR adjustment P
Maintained normal	1 (reference)			1 (reference)			1 (reference)			1 (reference)			1 (reference)		
Increased	1.05 (0.83–1.34)	0.684	0.77	0.78 (0.51–1.19)	0.245	0.4	0.84 (0.48–1.46)	0.533	0.998	1.36 (1.14–1.63)	0.001	0.002	1.13 (0.97–1.32)	0.111	0.333
Reversed	1 (0.71–1.42)	0.982	0.982	1.51 (0.97–2.36)	0.068	0.378	1.06 (0.52–2.15)	0.878	0.999	0.99 (0.74–1.33)	0.935	0.909	0.79 (0.61–1.03)	0.08	0.333
Persistent High	1.08 (0.91–1.29)	0.387	0.619	1.02 (0.77–1.34)	0.914	0.914	0.91 (0.62–1.34)	0.639	0.873	1.32 (1.15–1.51)	<0.001	0.001	1.06 (0.94–1.19)	0.334	0.752
Pre-DM															
Maintained normal	1 (reference)			1 (reference)			1 (reference)			1 (reference)			1 (reference)		
Increased	1.65 (0.87–3.13)	0.127	0.572	1.26 (0.38–4.21)	0.705	0.793	0 (0 - Inf)	0.998	0.998	1.21 (0.62–2.34)	0.576	0.718	0.93 (0.55–1.58)	0.797	0.945
Reversed	1.43 (0.61–3.35)	0.413	0.619	2.72 (0.87–8.43)	0.084	0.378	0 (0 - Inf)	0.998	0.999	1.22 (0.53–2.81)	0.638	0.718	1.05 (0.54–2.02)	0.893	0.945
Persistent High	1.27 (0.77–2.1)	0.34	0.619	0.73 (0.3–1.81)	0.499	0.642	0.56 (0.2–1.55)	0.264	0.792	1.53 (0.98–2.39)	0.062	0.186	1.03 (0.72–1.47)	0.879	0.945
DM															
Maintained normal	1 (reference)			1 (reference)			1 (reference)			1 (reference)			1 (reference)		
Increased	1.27 (0.48–3.39)	0.633	0.77	5.62 (0.56–56.18)	0.142	0.4	1.86 (0.11–30.72)	0.665	0.998	0.56 (0.12–2.69)	0.472	0.714	0.81 (0.16–4.09)	0.797	0.945
Reversed	0.49 (0.13–1.86)	0.294	0.619	4.6 (0.41–51.75)	0.217	0.4	0 (0 - Inf)	0.999	0.999	2.38 (0.79–7.15)	0.123	0.277	2.82 (0.86–9.32)	0.089	0.333
Persistent High	0.51 (0.23–1.13)	0.096	0.572	3.23 (0.41–25.49)	0.267	0.4	1.2 (0.12–11.66)	0.873	0.873	1.35 (0.59–3.12)	0.476	0.714	1.04 (0.38–2.79)	0.945	0.945

### Obesity indicators and brain structure in different glucose metabolic states

In individuals with NGR, higher BMI was significantly associated with lower grey matter volume (*β* = −837.29) and total brain volume (*β* = −630.32), but with increased white matter volume (*β* = 206.98) and WMHs volume (*β* = 96.51). WC was associated with a reduction in grey matter volume (*β* = −435.96) and total brain volume (*β* = −441.04). General, abdominal, and high body fat obesity were consistently linked to lower grey matter and total brain volumes and higher WMHs volume. Among individuals with prediabetes, similar patterns were observed. BMI was negatively associated with grey matter volume (*β* = −1076.42) and total brain volume (*β* = −1082.11), with no significant association with white matter volume. WC and BFP were also inversely related to both grey matter and total brain volumes. Notably, BFP showed a significant negative association with thalamus volume (*β* = −10.76). General and abdominal obesity remained significantly associated with reduced grey matter and total brain volumes. High body fat percentage was associated with marked reductions in grey matter (*β* = −6586.42), total brain volume (*β* = −7927.99), and thalamus volume (*β* = −112.29). In individuals with diabetes, the associations were more pronounced. BMI was negatively associated with grey matter (*β* = −1330.09) and total brain volumes (*β* = −1398.86). BFP was significantly associated with reduced thalamus (*β* = −18.32) and hippocampus (*β* = −12.5) volumes. High body fat percentage was particularly associated with decreased grey matter (*β* = −14230.44), total brain volume (*β* = −18844.27), thalamus (*β* = −261.47), and hippocampus volumes (*β* = −208.31; Supplementary Table S3).

### Change in obesity indicators and brain structure in different glucose metabolic states

Changes in BMI were significantly associated with alterations in brain structure across different glycemic statuses. Among individuals with NGR, both weight gain and persistent obesity were linked to reduced grey matter and total brain volumes, as well as increased WMHs volumes (e.g., persistent obesity: grey matter *β* = −8240.57; WMHs *β* = 1142.39). In the prediabetic group, only persistent obesity showed significant associations, with reduced grey matter (*β* = −12288.90), reduced total brain volume (*β* = −11956.18), and increased WMHs (*β* = 880.52). Among those with diabetes, persistent obesity was significantly associated with reductions in grey matter (*β* = −15052.73) and total brain volume (*β* = −14768.87), while weight gain was also linked to increased WMHs volume (*β* = 3332.76; Supplementary Table S4).

Changes in waist circumference were associated with brain structural alterations across glycemic statuses. In non-diabetic individuals, both incident and persistent abdominal obesity were significantly linked to lower grey and total brain volumes and greater WMHs volumes (e.g., persistent abdominal obesity: grey matter *β* = −10458.02; WMHs *β* = 1171.27). In the prediabetic group, persistent abdominal obesity was associated with reduced grey matter (*β* = −10710.78), lower total brain volume (*β* = −9919.20), and increased WMHs (*β* = 1040.56). Among individuals with diabetes, only persistent abdominal obesity showed significant associations with reduced grey (*β* = −12567.31) and total brain volume (*β* = −20084.49; Supplementary Table S5).

Changes in body fat percentage were associated with brain structural differences across glycemic statuses. In non-diabetic individuals, both increased and persistent high body fat were significantly associated with lower grey matter volume (e.g., persistent high: *β* = −4131.72) and higher WMHs volume (*β* = 621.38). Increased body fat was also linked to smaller thalamus and hippocampus volumes. In the prediabetic group, persistent high body fat was associated with lower grey and total brain volumes. Interestingly, reversed body fat in this group was related to significantly lower white matter and total brain volumes. Among individuals with diabetes, only persistent high body fat showed significant associations with reduced grey (*β* = −12876.12), total brain (*β* = −18301.25), thalamus (*β* = −274.18), and hippocampus volumes (*β* = −200.14; Supplementary Table S6).

## Discussion

In this large, population-based cohort study, we comprehensively examined the associations between various obesity indicators—including BMI, waist circumference, and body fat percentage—and both neuropsychiatric outcomes and brain structural measures across different glycemic statuses.

First, obesity indicators were consistently associated with higher risks of depression and anxiety, particularly among individuals with NGR. These associations were robust across different adiposity measures, including general, abdominal, and high body fat-defined obesity.

Interestingly, we also observed an inverse association between BMI and dementia risk in non-diabetic individuals. This counterintuitive finding has been reported previously and may reflect reverse causality or the influence of preclinical weight loss during the prodromal phase of dementia (21). Specifically, several prospective studies have shown that unintentional weight loss often precedes the clinical onset of dementia by years, suggesting that declining BMI may be a marker rather than a cause of disease risk (22, 23). Therefore, the apparent protective effect of higher BMI should be interpreted with caution, as it likely reflects the impact of prodromal disease processes rather than a true biological benefit. Conversely, abdominal obesity was positively associated with Parkinson’s disease risk in non-diabetic individuals, which may reflect a role for visceral adiposity in neurodegeneration through chronic inflammation and oxidative stress pathways (24).

In the prediabetic group, associations with depression and anxiety persisted but appeared slightly attenuated. Importantly, in individuals with diabetes, most associations with neurological outcomes (e.g., stroke, dementia, Parkinson’s disease) were no longer significant. However, the associations with depression and anxiety remained strong, suggesting that psychological burden in diabetes may be more closely linked to metabolic control, disease burden, or inflammatory responses than to body fat distribution alone (25, 26).

Dynamic changes in obesity status were also informative. Among individuals with normal glucose metabolism, both weight gain and persistent obesity were associated with increased risks of depression and anxiety, reinforcing the notion that sustained or worsening adiposity can negatively impact mental well-being (27). Even weight loss was linked to an elevated depression risk, which may reflect underlying illness or unintentional weight reduction, highlighting the complexity of interpreting weight changes in observational data (28).

Changes in abdominal obesity showed similar patterns: both incident and persistent abdominal obesity were associated with higher risks of depression and anxiety, while reversed abdominal obesity was also linked to depression. Notably, these associations were not observed in the diabetes group, possibly due to the overriding influence of diabetes-related comorbidities or smaller sample sizes limiting statistical power. Overall, these results emphasize the need for weight management strategies even among metabolically “normal” individuals to promote neuropsychiatric health.

Our neuroimaging findings further support a detrimental impact of obesity on brain structure, particularly on grey matter and total brain volumes (29). These associations were most prominent in individuals with diabetes, where higher BMI and BFP were consistently linked to reduced brain tissue volumes and increased white matter hyperintensities (WMHs)—markers of small vessel disease and brain aging. Importantly, body fat percentage in diabetes showed the most robust and consistent signal, with significant reductions in grey matter, thalamus, and hippocampus volumes alongside greater WMHs burden. This highlights body fat percentage as a particularly sensitive marker of neurostructural vulnerability in the context of hyperglycemia, and underscores the importance of monitoring fat composition rather than relying solely on BMI.

Similar but weaker patterns were observed in prediabetic individuals, while non-diabetics also showed reductions in grey matter and increases in WMHs with higher adiposity. These findings suggest that obesity may accelerate brain aging processes and structural degeneration, with more pronounced effects in the context of impaired glucose metabolism (30, 31). The synergistic impact of adiposity and hyperglycemia on the brain may operate via vascular, inflammatory, or insulin-resistance-related mechanisms (32).

Longitudinal changes in obesity status were also related to brain structure. Persistent obesity and abdominal obesity were consistently associated with lower grey matter and higher WMHs volumes across glycemic strata. Weight gain and increased abdominal girth were particularly detrimental in non-diabetic and prediabetic individuals, while persistent high body fat percentage showed the most pronounced associations with structural brain deficits in individuals with diabetes. These results suggest that both the presence and persistence of excess adiposity contribute to neurodegenerative changes and emphasize the importance of long-term weight management for brain health preservation.

All of these associations may be explained by several interrelated biological mechanisms: (1) Obesity, particularly visceral fat accumulation, is known to promote a pro-inflammatory state. Adipose tissue secretes cytokines such as IL-6, TNF-*α*, and CRP, which can cross the blood–brain barrier and contribute to neuroinflammation (33). This inflammatory environment may impair neurogenesis, accelerate neurodegeneration, and alter neurotransmitter systems involved in mood regulation, thereby increasing the risk of depression and anxiety (34). (2) In both prediabetes and diabetes, insulin resistance compromises glucose delivery to neurons, potentially leading to brain energy deficits, oxidative stress, and synaptic dysfunction (35). These metabolic disturbances may explain the stronger associations observed between adiposity and brain atrophy or white matter lesions in hyperglycemic populations. (3) Obesity is a well-established risk factor for hypertension, dyslipidemia, and atherosclerosis, all of which contribute to cerebral small vessel disease (36). This is supported by our finding that high adiposity is associated with increased WMH volumes, a marker of microvascular brain injury and cognitive decline. (4) Adiposity may also alter HPA axis function, leading to chronic cortisol elevation (37). This hormonal imbalance can negatively affect mood and hippocampal integrity, contributing to both depression and structural brain changes (38). (5) Lower levels of BDNF, often observed in individuals with obesity and metabolic syndrome, may also mediate the link between adiposity and brain atrophy, especially in regions critical for cognition and emotion (39). Future research integrating inflammatory biomarker profiles, advanced neuroimaging, and longitudinal neuropsychiatric assessments will be essential to elucidate the mediating role of neuroinflammation in the complex interplay among adiposity, glycemic status, and brain health outcomes.

Overall, our findings suggest that obesity—particularly persistent or increasing adiposity—adversely affects both mental health and brain structure, and these associations are modified by glycemic status. Early and sustained interventions targeting weight management may play a critical role in preventing neuropsychiatric disorders and mitigating brain aging, especially in individuals at risk of or living with metabolic dysfunction.

The strengths of our study include a large, well-characterized cohort, prospective design, rich covariate adjustment, and high-quality MRI data. However, several limitations merit consideration. First, the observational nature of the study precludes causal inference. Second, residual confounding and measurement errors cannot be fully excluded. Third, as 95% of participants in the UK Biobank are White and middle-aged, the findings may not be generalizable to younger individuals or more ethnically diverse populations. This limitation is particularly important given the global burden of obesity and diabetes, and the fact that adiposity distribution, metabolic risk, and susceptibility to neuropsychiatric disorders differ across ethnic groups. Future studies in more ethnically diverse cohorts are warranted to determine whether these associations hold across different genetic and sociocultural contexts. Fourth, the neuroimaging analyses were based on cross-sectional MRI data, and the absence of longitudinal imaging precludes assessment of temporal changes in brain structure. Future studies with repeated neuroimaging measures are warranted to clarify the trajectory of structural alterations associated with adiposity and glycemic status.

## Conclusion

Our findings suggest that higher and sustained adiposity, particularly in the context of impaired glucose metabolism, adversely affects both mental health and brain structure. These effects may be driven by a combination of inflammatory, metabolic, vascular, and neuroendocrine mechanisms. Preventive strategies aimed at controlling weight and metabolic risk may have broader benefits for preserving neuropsychiatric and cognitive health across the lifespan.

## Data Availability

The original contributions presented in the study are included in the article/Supplementary material, further inquiries can be directed to the corresponding author.

## Footnotes

^1^ www.ukbiobank.ac.uk

^2^ https://biobank.ndph.ox.ac.uk/showcase/showcase/docs/brain_mri.pdf
