# Supplementary material for: Association between changes in obesity status and neuropsychiatric health and brain structure in different glucose status
Source: Front Nutr. 2025 Oct 3;12:1676168. doi: 10.3389/fnut.2025.1676168 (PMC12531048; doi:10.3389/fnut.2025.1676168)

Table S1. Baseline characteristics of the subjects

Characteristics	All	stroke	dementia	Parkinson's disease	depression	anxiety
Sample size	423750	11975	8478	2940	18037	21631
Age, median (IQR)	58.00 [50.00, 63.00]	63.00 [58.00, 67.00]	65.00 [62.00, 68.00]	64.00 [60.00, 67.00]	57.00 [49.00, 63.00]	58.00 [49.00, 63.00]
Ethnicity, n (%)						
White	402288 (95.0)	11448 (95.7)	8173 (96.6)	2841 (96.7)	17194 (95.5)	20615 (95.4)
Black	10656 (2.5)	269 (2.2)	135 (1.6)	47 (1.6)	355 (2.0)	480 (2.2)
Asian	3187 (0.8)	90 (0.8)	39 (0.5)	10 (0.3)	91 (0.5)	114 (0.5)
Other	7176 (1.7)	158 (1.3)	116 (1.4)	39 (1.3)	371 (2.1)	390 (1.8)
Hypertension, n (%)	108701 (25.7)	5054 (42.2)	3594 (42.4)	1086 (36.9)	5840 (32.4)	6434 (29.7)
High cholesterol, n (%)	48456 (11.4)	2122 (17.7)	1793 (21.1)	554 (18.8)	2449 (13.6)	2675 (12.4)
Glucose metabolic states, n (%)						
NGR	293095 (84.3)	7614 (76.8)	5229 (75.0)	1906 (79.8)	12080 (82.4)	14584 (83.4)
Pre-DM	41261 (11.9)	1521 (15.3)	1141 (16.4)	344 (14.4)	1787 (12.2)	2209 (12.6)
DM	13483 (3.9)	781 (7.9)	600 (8.6)	139 (5.8)	796 (5.4)	689 (3.9)

Townsend deprivation index median (IQR)	-2.20 [-3.68, 0.41]	-1.99 [-3.53, 0.90]	-1.93 [-3.56, 1.01]	-2.38 [-3.77, 0.23]	-1.51 [-3.29, 1.64]	-1.86 [-3.50, 1.11]
Smoking, n (%)						
Never	234617 (55.6)	5492 (46.2)	3905 (46.5)	1527 (52.3)	8382 (46.8)	11042 (51.4)
Former	145328 (34.5)	4714 (39.6)	3617 (43.1)	1215 (41.6)	6528 (36.5)	7632 (35.5)
Current	41688 (9.9)	1689 (14.2)	877 (10.4)	177 (6.1)	2986 (16.7)	2807 (13.1)
Alcohol status, n (%)						
Never	18247 (4.3)	626 (5.2)	545 (6.5)	143 (4.9)	913 (5.1)	1147 (5.3)
Former	13066 (3.1)	499 (4.2)	465 (5.5)	137 (4.7)	1057 (5.9)	998 (4.6)
Current	391449 (92.6)	10827 (90.6)	7431 (88.0)	2650 (90.4)	15986 (89.0)	19415 (90.1)
Sleep time, n (%)						
Short	101675 (24.2)	3079 (25.9)	2119 (25.4)	655 (22.5)	5535 (31.1)	6187 (28.9)
Normal	313288 (74.4)	8485 (71.5)	5961 (71.4)	2184 (74.9)	11739 (66.0)	14752 (68.9)
Long	5979 (1.4)	308 (2.6)	263 (3.2)	76 (2.6)	524 (2.9)	461 (2.2)
Sedentary behaviors(h/week)	5.00 [4.00, 6.00]	5.00 [4.00, 6.00]	5.00 [4.00, 6.00]	5.00 [4.00, 6.00]	5.00 [4.00, 6.00]	5.00 [4.00, 6.00]
MET (h/week)	30.37 [13.97, 59.80]	30.20 [13.11, 63.55]	31.50 [13.70, 67.55]	28.92 [13.32, 57.23]	28.17 [11.62, 60.20]	29.77 [12.83, 61.83]

Body mass index, kg/m2	26.66 [24.10, 29.76]	27.40 [24.76, 30.59]	27.09 [24.38, 30.18]	27.10 [24.60, 29.94]	27.55 [24.57, 31.21]	26.89 [24.12, 30.34]
Waist circumference, median (IQR), cm	90.00 [80.00, 99.00]	94.00 [85.00, 102.00]	92.00 [83.00, 101.00]	94.00 [85.00, 101.50]	91.00 [82.00, 101.00]	89.00 [80.00, 99.00]
Body fat percentage, median (IQR), %	30.70 [24.90, 37.40]	30.40 [25.00, 37.00]	31.00 [25.40, 37.50]	29.30 [24.48, 36.20]	33.30 [26.90, 39.90]	33.30 [26.90, 39.50]
General obesity, n (%)	100008 (23.6)	3449 (28.8)	2219 (26.2)	720 (24.5)	5739 (31.8)	5800 (26.8)
Abdominal obesity, n (%)a	137848 (32.5)	4786 (40.0)	3255 (38.4)	1069 (36.4)	7692 (42.6)	8117 (37.5)
High body fat percentage, n (%)	237818 (56.1)	7582 (63.3)	5363 (63.3)	1823 (62.0)	11574 (64.2)	13154 (60.8)

NGR, Normal glucose regulation; Pre-DM, Prediabetes mellitus; DM, Diabetes mellitus.

Table S2. Obesity indicators and neuropsychiatric health in different glucose metabolic states

	stroke			dementia			Parkinson’s disease			depression			anxiety		
NGR	HR (95%CI	P	FDR adjustment P	HR (95%CI	P	FDR adjustment P	HR (95%CI	P	FDR adjustment P	HR (95%CI	P	FDR adjustment P	HR (95%CI	P	FDR adjustment P
BMI, per 1-unit increase	1.01(1 - 1.02)	0.001	0.002	0.98(0.97 - 0.99)	<0.001	<0.001	1(0.99 - 1.02)	0.463	0.463	1.03(1.03 - 1.04)	<0.001	<0.001	1.01(1 - 1.01)	<0.001	<0.001
WC, per 1-unit increase	1(1 - 1.01)	<0.001	<0.001	1(0.99 - 1)	0.053	0.08	1.01(1 - 1.01)	0.005	0.03	1.01(1.01 - 1.02)	<0.001	<0.001	1.01(1 - 1.01)	<0.001	<0.001
BFP, per 1-unit increase	1(1 - 1.01)	0.026	0.026	0.99(0.98 - 0.99)	<0.001	<0.001	1(1 - 1.01)	0.402	0.463	1.02(1.02 - 1.03)	<0.001	<0.001	1.01(1.01 - 1.01)	<0.001	<0.001
General obesity	1.1(1.04 -	0.001	0.002	0.97(0.9 -	0.347	0.416	1.05(0.93 -	0.436	0.463	1.33(1.27 -	<0.001	<0.001	1.11(1.06 -	<0.001	<0.001

	1.17)			1.04)			1.18)			1.39)			1.16)		
Abdominal obesity	1.09(1.03 - 1.15)	0.002	0.002	0.99(0.93 - 1.06)	0.824	0.824	1.11(1 - 1.24)	0.042	0.053	1.32(1.27 - 1.38)	<0.001	<0.001	1.1(1.06 - 1.15)	<0.001	<0.001
High body fat percentage	1.08(1.04 - 1.13)	<0.001	<0.001	0.91(0.85 - 0.96)	0.001	0.002	1.04(0.94 - 1.16)	0.398	0.398	1.26(1.21 - 1.32)	<0.001	<0.001	1.12(1.07 - 1.16)	<0.001	<0.001
Pre-DM															
BMI, per 1-unit increase	1.02(1.01 - 1.03)	0.001	0.002	1(0.98 - 1.01)	0.863	0.896	1.01(0.99 - 1.04)	0.269	0.448	1.03(1.02 - 1.04)	<0.001	<0.001	1(0.99 - 1.01)	0.896	0.896
WC, per 1-unit increase	1.01(1 - 1.01)	0.001	0.002	1(0.99 - 1.01)	0.894	0.894	1.01(1 - 1.02)	0.151	0.189	1.02(1.01 - 1.02)	<0.001	<0.001	1(1 - 1.01)	<0.001	<0.001
BFP, per 1-unit	1.01(1 - 1.02)	0.059	0.071	0.99(0.98 - 1)	0.24	0.4	1.01(0.99 -	0.366	0.458	1.03(1.02 -	<0.001	<0.001	1(0.99 - 1.01)	0.953	0.953

increase							1.03)			1.04)					
General obesity	1.24(1.12 - 1.36)	<0.001	<0.001	1.02(0.89 - 1.17)	0.781	0.921	1.13(0.88 - 1.45)	0.331	0.552	1.34(1.2 - 1.51)	<0.001	<0.001	0.99(0.89 - 1.11)	0.921	0.921
Abdominal obesity	1.18(1.05 - 1.33)	0.004	0.006	1.03(0.9 - 1.17)	0.69	0.69	1.19(0.94 - 1.5)	0.16	0.2	1.38(1.23 - 1.55)	<0.001	<0.001	1.22(1.15 - 1.3)	<0.001	<0.001
High body fat percentage	1.02(0.9 - 1.15)	0.797	0.806	0.98(0.85 - 1.13)	0.806	0.806	1.25(0.94 - 1.66)	0.123	0.308	1.26(1.11 - 1.42)	<0.001	<0.001	1.03(0.93 - 1.15)	0.536	0.806
DM															
BMI, per 1-unit increase	0.99(0.98 - 1.01)	0.519	0.883	1.01(0.99 - 1.03)	0.409	0.511	1.02(0.98 - 1.06)	0.263	0.438	1.03(1.01 - 1.04)	0.001	0.002	1.03(1.02 - 1.05)	<0.001	<0.001
WC, per 1-unit	1(0.99 - 1.01)	0.894	0.894	1.01(1 - 1.01)	0.068	0.113	1.01(0.99 -	0.225	0.281	1.01(1.01 -	<0.001	<0.001	1.02(1.01 -	<0.001	<0.001

increase							1.02)			1.02)			1.02)		
BFP, per 1-unit increase	0.99(0.98 - 1.01)	0.437	0.65	1(0.98 - 1.02)	0.937	0.937	1.01(0.98 - 1.04)	0.52	0.65	1.02(1.01 - 1.04)	0.003	0.007	1.03(1.02 - 1.05)	<0.001	<0.001
General obesity	0.96(0.81 - 1.13)	0.589	0.736	1.06(0.88 - 1.27)	0.566	0.736	1.01(0.69 - 1.46)	0.975	0.975	1.27(1.07 - 1.51)	0.007	0.018	1.43(1.18 - 1.72)	<0.001	<0.001
Abdominal obesity	1.02(0.86 - 1.2)	0.842	0.842	1.18(0.97 - 1.43)	0.092	0.153	1.33(0.91 - 1.94)	0.144	0.18	1.37(1.14 - 1.65)	0.001	0.005	1.37(1.12 - 1.67)	0.002	0.005
High body fat percentage	0.88(0.73 - 1.06)	0.185	0.231	1.02(0.81 - 1.28)	0.856	0.856	1.41(0.85 - 2.32)	0.179	0.231	1.26(1.11 - 1.42)	<0.001	<0.001	1.27(1 - 1.6)	0.049	0.049

NGR, Normal glucose regulation; Pre-DM, Prediabetes mellitus; DM, Diabetes mellitus; BMI, body mass index; WC, waist circumference; BFP, body fat percentage

Table S3. Obesity indicators and brain structure in different glucose metabolic states

	Grey matter			White matter			Total brain			Volume of thalamus			Volume of hippocampus			Volume of white matter hyperintensities		
	β coefcients (95%CI)	P	FDR adjustment P	β coefcients (95%CI)	P	FDR adjustment P	β coefcients (95%CI)	P	FDR adjustment P	β coefcients (95%CI)	P	FDR adjustment P	β coefcients (95%CI)	P	FDR adjustment P	β coefcients (95%CI)	P	FDR adjustment P
NGR																		
BMI, per 1-unit increase	-837.29(-957.78 to -716.81)	<0.001	<0.001	206.98(75.92 to 338.04)	0.002	0.012	-630.32(-833.78 to -426.87)	<0.001	<0.001	-0.8(-5.03 to 3.44)	0.712	0.782	2.15(-0.64 to 4.94)	0.131	0.418	96.51(75.59 to 117.43)	<0.001	<0.001
WC, per 1-unit increase	-435.96(-482.51 to -389.42)	<0.001	<0.001	-5.08(-55.88 to 45.72)	0.845	0.845	-441.04(-519.75 to -362.33)	<0.001	<0.001	2.07(0.43 to 3.71)	0.013	0.039	0.7(-0.38 to 1.78)	0.206	0.418	42.34(34.25 to 50.44)	<0.001	<0.001
BFP, per 1-unit increase	-358.28(-439.21 to -277.36)	<0.001	<0.001	119.1(31.28 to 206.93)	0.008	0.024	-239.19(-375.6 to -102.78)	0.001	<0.001	-5.05(-7.89 to -2.22)	<0.001	<0.001	-0.95(-2.82 to 0.92)	0.318	0.55	47.7(33.7 to 61.71)	<0.001	<0.001
Gener	-7108.96(-83	<0.0	<0.001	1504.99(104	0.0	0.070	-5603.99(-77	<0.0	<0.001	-34.87(-80	0.13	0.295	12.86(-16.	0.3	0.55	802.14(57	<0.0	<0.001

al obesity	98.08 to -5819.84)	01		.63 to 2905.34)	35		78.22 to -3429.77)	01		.08 to 10.34)	1		93 to 42.66)	97		8.56 to 1025.73)	01	
Abdominal obesity	-7817.31(-8954.26 to -6680.37)	<0.001	<0.001	775.62(-461.19 to 2012.43)	0.219	0.263	-7041.75(-8960.87 to -5122.63)	<0.001	<0.001	25.55(-14.38 to 65.49)	0.21	0.42	2.71(-23.61 to 29.03)	0.84	0.84	777.39(580 to 974.78)	<0.001	<0.001
High body fat percentage	-3518.18(-4472.25 to -2564.1)	<0.001	<0.001	1010.43(-24.58 to 2045.44)	0.056	0.084	-2507.77(-4115.27 to -900.27)	0.002	<0.001	-58.43(-91.84 to -25.02)	0.001	0.003	-20.49(-48.62 to 7.64)	0.153	0.229	379.1(213.92 to 544.27)	<0.001	<0.001
Pre-DM																		
BMI, per 1-unit increase	-1076.42(-1401.15 to -751.68)	<0.001	<0.001	-5.69(-359.7 to 348.32)	0.975	0.975	-1082.11(-1622.26 to -541.96)	<0.001	<0.001	-1.82(-13.3 to 9.66)	0.756	0.782	4.92(-2.57 to 12.41)	0.198	0.418	47.33(-13.06 to 107.72)	0.124	0.203
WC, per 1-unit increase	-490.97(-619.02 to -362.92)	<0.001	<0.001	-63.8(-203.74 to 76.14)	0.371	0.648	-554.77(-767.89 to -341.64)	<0.001	<0.001	1.53(-3.01 to 6.07)	0.509	0.549	1.18(-1.78 to 4.14)	0.435	0.559	31.82(8.08 to 55.55)	0.009	0.023
BFP,	-538.92(-779.	<0.0	<0.001	33.97(-227.6	0.7	0.951	-504.95(-904.	0.01	<0.001	-10.76(-19	0.01	0.016	1.46(-4.08	0.6	0.681	5.9(-38.65	0.79	0.843

per 1-unit increase	94 to -297.9)	01		5 to 295.58)	99		84 to -105.07)	3		.04 to -2.48)	1		to 7)	05		to 50.44)	5	
General obesity	-10495.15(-13768.31 to -7221.99)	<0.001	<0.001	417.82(-3148.57 to 3984.22)	0.818	0.951	-10077.25(-15521.22 to -4633.27)	<0.001	<0.001	-39.73(-154.44 to 74.98)	0.497	0.705	-26.08(-101.55 to 49.4)	0.498	0.598	496.71(-110.64 to 1104.05)	0.109	0.196
Abdominal obesity	-7846.51(-10868.77 to -4824.24)	<0.001	<0.001	163.67(-3121.02 to 3448.37)	0.922	0.975	-7682.83(-12700.68 to -2664.98)	0.003	<0.001	15.03(-91.53 to 121.6)	0.782	0.782	12.06(-57.5 to 81.62)	0.734	0.777	462.46(-95.99 to 1020.92)	0.105	0.196
High body fat percentage	-6586.42(-9441.46 to -3731.38)	<0.001	<0.001	-1341.48(-4440.84 to 1757.89)	0.396	0.648	-7927.99(-12661.79 to -3194.2)	0.001	<0.001	-112.29(-211.18 to -13.41)	0.026	0.026	29.88(-35.71 to 95.46)	0.372	0.372	38.81(-487.84 to 565.47)	0.885	0.885
DM																		
BMI, per 1-unit increase	-1330.09(-1924.3 to -735.88)	<0.001	<0.001	-68.78(-734.71 to 597.14)	0.839	0.951	-1398.86(-2386.17 to -411.54)	0.006	<0.001	-7.69(-28.42 to 13.03)	0.466	0.705	-8.44(-21.63 to 4.75)	0.209	0.418	92.26(-34.63 to 219.15)	0.154	0.231
WC, per	-556.52(-790.08 to -322.96)	<0.001	<0.001	-131.87(-393.96 to	0.323	0.646	-688.39(-1075.87 to	0.001	<0.001	-2.49(-10.66 to 5.67)	0.549	0.549	-4.76(-9.98 to 0.46)	0.074	0.333	40.27(-8.88 to 89.43)	0.108	0.196

1-unit increase				130.21)			-300.92)											
BFP, per 1-unit increase	-1180.65(-1657.88 to -703.42)	<0.001	<0.001	-297.37(-833.53 to 238.79)	0.276	0.621	-1478.02(-2270 to -686.04)	<0.001	<0.001	-18.32(-34.97 to -1.67)	0.031	0.046	-12.5(-23.09 to -1.9)	0.021	0.042	70.04(-30.01 to 170.09)	0.17	0.235
General obesity	-11442.76(-17858.24 to -5027.27)	<0.001	<0.001	1049.15(-6099.29 to 8197.58)	0.773	0.951	-11053.66(-21560.85 to -546.46)	0.039	<0.001	-131.45(-351.7 to 88.8)	0.242	0.436	-137.55(-278.88 to 3.79)	0.056	0.333	286.55(-1043.7 to 1616.8)	0.672	0.806
Abdominal obesity	-9002.16(-15231.06 to -2773.26)	0.005	<0.001	-4875.32(-11781.35 to 2030.71)	0.166	0.563	-13877.52(-24138.41 to -3616.63)	0.008	<0.001	-31.06(-246.45 to 184.33)	0.777	0.782	-63.7(-200.77 to 73.37)	0.362	0.550	480.78(-799.75 to 1761.31)	0.461	0.593
High body fat percentage	-14230.44(-20604.15 to -7856.74)	<0.001	<0.001	-4613.6(-11746.62 to 2519.42)	0.204	0.563	-18844.27(-29394.18 to -8294.37)	<0.001	<0.001	-261.47(-482.91 to -40.03)	0.021	0.026	-208.31(-348.96 to -67.66)	0.004	0.012	173.88(-1143.61 to 1491.38)	0.796	0.843

NGR, Normal glucose regulation; Pre-DM, Prediabetes mellitus; DM, Diabetes mellitus; BMI, body mass index; WC, waist circumference; BFP, body fat percentage; FDR, false discovery rate

Table S4. Association of changes in weight and brain structure in different glucose metabolic states

	Grey matter			White matter			total brain			Volume of thalamus			Volume of hippocampus			Volume of white matter hyperintensities		
	β coefcients (95%CI)	P	FDR adjustment P	β coefcients (95%CI)	P	FDR adjustment P	β coefcients (95%CI)	P	FDR adjustment P	β coefcients (95%CI)	P	FDR adjustment P	β coefcients (95%CI)	P	FDR adjustment P	β coefcients (95%CI)	P	FDR adjustment P
NGR																		
Maintained normal	1 (reference)			1 (reference)			1 (reference)			1 (reference)			1 (reference)			1 (reference)		
Weight gain	-6725.52(-9041.46 to -4409.58)	<0.001	<0.001	1360.5(-1163.94 to 3884.94)	0.291	0.349	-5365.12(-9280.33 to -1449.91)	0.007	0.014	-57.36(-138.83 to 24.1)	0.168	0.252	-1.69(-55.41 to 52.03)	0.951	0.951	851.86(449.83 to 1253.89)	<0.001	<0.001
Weight loss	-3840.03(-6306.21 to -1373.86)	0.002	0.012	-1483.65(-4171.84 to 1204.54)	0.279	0.419	-5323.73(-9492.92 to -1154.55)	0.012	0.036	33.53(-53.22 to 120.28)	0.449	0.539	42.27(-14.93 to 99.48)	0.147	0.294	-70.27(-498.17 to 357.63)	0.748	0.748
Persistent obesity	-8240.57(-9705.73 to -6775.41)	<0.001	<0.001	2642.38(1045.32 to 4239.44)	0.001	0.002	-5598.19(-8075.11 to -3121.27)	<0.001	<0.001	-53.66(-105.2 to -2.12)	0.041	0.049	3.7(-30.29 to 37.68)	0.831	0.831	1142.39(887.52 to 1397.26)	<0.001	<0.001
Pre-DM																		
Maintai	1 (reference)			1			1 (reference)			1			1			1		

ned normal				(reference)						(reference)			(reference)			(reference)		
Weight gain	-5392.11(-11969.98 to 1185.76)	0.108	0.476	2476.71(-4715.85 to 9669.28)	0.5	0.722	-2915.47(-13881.91 to 8050.97)	0.602	0.722	23.46(-209.74 to 256.65)	0.844	0.844	91.83(-60.64 to 244.3)	0.238	0.476	766.85(-428.24 to 1961.94)	0.208	0.476
Weight loss	-5773.88(-11655.63 to 107.87)	0.054	0.324	2228.32(-4203.07 to 8659.72)	0.497	0.529	-3545.45(-13351.34 to 6260.43)	0.478	0.529	113.42(-95.1 to 321.93)	0.286	0.529	51.98(-84.35 to 188.31)	0.455	0.529	-344.95(-1419.97 to 730.06)	0.529	0.529
Persistent obesity	-12288.9(-15989.3 to -8588.51)	<0.001	<0.001	332.63(-3713.57 to 4378.82)	0.872	0.872	-11956.18(-18125.37 to -5786.98)	<0.001	<0.001	-55.72(-187.03 to 75.59)	0.405	0.486	-40.14(-126 to 45.71)	0.359	0.486	880.52(198.54 to 1562.51)	0.011	0.022
DM																		
Maintained normal	1 (reference)			1 (reference)			1 (reference)			1 (reference)			1 (reference)			1 (reference)		
Weight gain	-16316.65(-31170.67 to -1462.64)	0.031	0.099	6607.06(-10294.55 to 23508.66)	0.443	0.58	-9708.88(-34659.17 to 15241.41)	0.445	0.58	-72.79(-591.56 to 445.98)	0.783	0.783	119.17(-214.49 to 452.83)	0.483	0.58	3332.76(268.01 to 6397.51)	0.033	0.099
Weight loss	-6535.93(-16937.19 to 3865.32)	0.218	0.435	3807.67(-8027.38 to 15642.72)	0.528	0.634	-2728.59(-20199.59 to 14742.41)	0.759	0.759	-270.23(-633.49 to 93.03)	0.145	0.435	-143.94(-377.58 to 89.7)	0.227	0.435	-1161.68(-3316.47 to 993.1)	0.29	0.435
Persistent	-15052.73(-22077.16 to	<0.001	<0.001	283.76(-7708.97 to	0.944	0.944	-14768.87(-26567.8 to	0.014	0.042	-48.28(-293.61 to	0.699	0.839	-115.2(-272.98 to	0.152	0.228	1260.61(-235.18 to	0.098	0.196

obesity	-8028.31)			8276.49)			-2969.94)			197.04)			42.59)			2756.39)		

NGR, Normal glucose regulation; Pre-DM, Prediabetes mellitus; DM, Diabetes mellitus;

Table S5. Changes in waist circumference and brain structure in different glucose metabolic states

	Grey matter			White matter			Total brain			Volume of thalamus			Volume of hippocampus			Volume of white matter hyperintensities		
	β coefcients (95%CI)	P	FDR adjustment P	β coefcients (95%CI)	P	FDR adjustment P	β coefcients (95%CI)	P	FDR adjustment P	β coefcients (95%CI)	P	FDR adjustment P	β coefcients (95%CI)	P	FDR adjustment P	β coefcients (95%CI)	P	FDR adjustment P
NGR																		
Maintained normal WC	1 (reference)			1 (reference)			1 (reference)			1 (reference)			1 (reference)			1 (reference)		
Incident abdominal obesity	-6188.05(-7784.5 to -4591.6)	<0.001	<0.001	-2188.46(-3933.11 to -443.81)	0.014	0.017	-8376.55(-11078.16 to -5674.93)	<0.001	<0.001	-87.26(-143.56 to -30.96)	0.002	0.003	-32.05(-69.19 to 5.08)	0.091	0.091	723.21(445.97 to 1000.45)	<0.001	<0.001
Reversed abdominal	-3777.86(-5714.02 to -1841.71)	<0.001	<0.001	-1567.41(-3683.3 to 548.49)	0.147	0.176	-5345.29(-8621.78 to -2068.81)	0.001	0.003	109.61(41.33 to 177.89)	0.002	0.004	43.47(-1.57 to 88.51)	0.059	0.088	230.95(-106.72 to 568.62)	0.18	0.18

obesity																		
Persistent abdominal obesity	-10458.02(-11790.53 to -9125.5)	<0.001	<0.001	1360.65(-95.56 to 2816.87)	0.067	0.1	-9097.44(-11352.41 to -6842.48)	<0.001	<0.001	-21.24(-68.22 to 25.74)	0.376	0.376	-22.22(-53.21 to 8.77)	0.16	0.192	1171.27(938.93 to 1403.61)	<0.001	<0.001
Pre-DM																		
Maintained normal WC	1 (reference)			1 (reference)			1 (reference)			1 (reference)			1 (reference)			1 (reference)		
Incident abdominal obesity	-7461.51(-12385.62 to -2537.4)	0.003	0.018	-580.29(-5964.85 to 4804.27)	0.833	0.833	-8041.6(-16248.29 to 165.1)	0.055	0.165	105.68(-68.84 to 280.2)	0.235	0.282	69.7(-44.31 to 183.71)	0.231	0.282	768.33(-126.45 to 1663.12)	0.092	0.184
Reversed abdominal obesity	-3116.92(-8287.72 to 2053.88)	0.237	0.628	-1307.42(-6961.74 to 4346.89)	0.65	0.856	-4424.14(-13041.98 to 4193.7)	0.314	0.628	-0.72(-183.97 to 182.54)	0.994	0.994	-22.46(-142.18 to 97.26)	0.713	0.856	-504.6(-1452.63 to 443.44)	0.297	0.628
Persist	-10710.78(-14	<0.0	<0.001	791.58(-297	0.6	0.68	-9919.2(-1565	0.00	0.002	41.85(-80.	0.5	0.602	40.78(-39	0.3	0.476	1040.56(4	0.00	0.002

ent abdomi nal obesity	152.25 to -7269.32)	01		1.69 to 4554.85)	8		4.86 to -4183.53)	1		33 to 164.02)	02		.04 to 120.6)	17		10.39 to 1670.74)	1	
DM																		
Maintai ned normal WC	1 (reference)			1 (reference)			1 (reference)			1 (reference)			1 (referenc e)			1 (reference)		
Inciden t abdomi nal obesity	-3938.8(-1507 8.71 to 7201.12)	0.48 8	0.732	-2981.49(-15 571.83 to 9608.84)	0.6 42	0.77	-6919.77(-254 52.58 to 11613.04)	0.46 4	0.732	180.41(-2 07.03 to 567.86)	0.3 61	0.732	21.43(-22 7.46 to 270.31)	0.8 66	0.866	879.94(-14 63.22 to 3223.1)	0.46 1	0.732
Revers ed abdomi nal obesity	-1133.02(-112 36.36 to 8970.32)	0.82 6	0.983	741.15(-106 77.65 to 12159.94)	0.8 99	0.983	-391.9(-17200 .22 to 16416.42)	0.96 3	0.983	-3.74(-35 5.14 to 347.65)	0.9 83	0.983	89.07(-13 6.66 to 314.79)	0.4 39	0.983	-617.73(-2 719.8 to 1484.34)	0.56 4	0.983
Persist ent abdomi nal	-12567.31(-19 457.49 to -5677.13)	<0.0 01	<0.001	-7517.26(-15 304.55 to 270.03)	0.0 58	0.116	-20084.49(-31 547.27 to -8621.7)	0.00 1	0.003	10.05(-22 9.59 to 249.69)	0.9 34	0.934	-99.58(-2 53.52 to 54.36)	0.2 04	0.245	1054.73(-4 01.31 to 2510.77)	0.15 5	0.232

obesity																		

NGR, Normal glucose regulation; Pre-DM, Prediabetes mellitus; DM, Diabetes mellitus;

Table S6. Changes in body fat percentage and brain structure in different glucose metabolic states

	Grey matter			White matter			Total brain			Volume of thalamus			Volume of hippocampus			Volume of white matter hyperintensities		
	β coefcients (95%CI)	P	FDR adjustment P	β coefcients (95%CI)	P	FDR adjustment P	β coefcients (95%CI)	P	FDR adjustment P	β coefcients (95%CI)	P	FDR adjustment P	β coefcients (95%CI)	P	FDR adjustment P	β coefcients (95%CI)	P	FDR adjustment P
NGR																		
Maintained normal	1 (reference)			1 (reference)			1 (reference)			1 (reference)			1 (reference)			1 (reference)		
Increased	-1541.22(-2983.7 to -98.75)	0.036	0.054	-805.31(-2375.94 to 765.32)	0.315	0.315	-2346.55(-4783.7 to 90.6)	0.059	0.071	-122.99(-173.72 to -72.26)	<0.001	<0.001	-37.73(-71.22 to -4.25)	0.027	0.054	450.24(200.9 to 699.58)	<0.001	<0.001
Reversed	-2326.52(-4405.05 to -247.99)	0.028	0.168	-492.97(-2756.16 to 1770.23)	0.669	0.753	-2819.55(-6331.37 to 692.26)	0.116	0.298	-23.64(-96.73 to 49.45)	0.526	0.631	-1.23(-49.48 to 47.01)	0.96	0.96	-64.31(-425.1 to 296.49)	0.727	0.77
Persistent High	-4131.72(-5217.97 to -3045.48)	<0.001	<0.001	1086.23(-96.53 to 2268.98)	0.072	0.072	-3045.52(-4880.8 to -1210.24)	0.001	0.002	-106.44(-144.64 to -68.24)	<0.001	<0.001	-38.13(-63.35 to -12.92)	0.003	0.04	621.38(433.42 to 809.33)	<0.001	<0.001
Pre-DM																		
Maintai	1 (reference)			1 (reference)			1 (reference)			1			1			1		

ned normal										(reference)			(reference)			(reference)		
Increased	-68.67(-4668.99 to 4531.66)	0.977	0.977	-468.24(-5500.63 to 4564.16)	0.855	0.977	-536.81(-8213.42 to 7139.79)	0.891	0.977	-89.8(-252.96 to 73.37)	0.281	0.977	-36.46(-143.14 to 70.22)	0.503	0.977	-40.87(-886.16 to 804.41)	0.924	0.977
Reversed	-5540.37(-11678.11 to 597.38)	0.077	0.115	-9114.23(-15828.45 to -2400.02)	0.008	0.024	-14654.69(-24896.8 to -4412.58)	0.005	0.024	-217.71(-435.37 to -0.04)	0.05	0.1	-120.9(-263.21 to 21.42)	0.096	0.115	-572.94(-1708.13 to 562.25)	0.322	0.322
Persistent High	-6803.26(-10067.36 to -3539.17)	<0.001	<0.001	-686.64(-4257.3 to 2884.03)	0.706	0.768	-7489.97(-12936.79 to -2043.15)	0.007	0.021	-83.32(-199.14 to 32.5)	0.158	0.316	46.11(-29.62 to 121.83)	0.233	0.35	90.38(-509.56 to 690.32)	0.768	0.768
DM																		
Maintained normal	1 (reference)			1 (reference)			1 (reference)			1 (reference)			1 (reference)			1 (reference)		
Increased	9216.42(-1580.63 to 20013.47)	0.094	0.564	-5251.3(-17603.04 to 7100.43)	0.404	0.668	3966.03(-14159.05 to 22091.11)	0.668	0.668	-152.61(-529.7 to 224.48)	0.427	0.668	-54.58(-296.46 to 187.3)	0.658	0.668	-576.48(-2895.53 to 1742.57)	0.626	0.668
Reversed	-8155.77(-21216.07 to 4904.52)	0.22	0.434	-5549.44(-20490.3 to 9391.43)	0.466	0.466	-13704.97(-35629.36 to 8219.42)	0.22	0.434	-246.56(-702.69 to 209.58)	0.289	0.434	-133.72(-426.3 to 158.86)	0.37	0.444	-1689.84(-4478.25 to 1098.56)	0.234	0.434
Persistent	-12876.12(-20245.05 to	0.001	0.006	-5425.08(-13855.09 to	0.207	0.248	-18301.25(-30671.53 to	0.004	0.012	-274.18(-531.54 to	0.037	0.055	-200.14(-365.22 to	0.018	0.036	189.97(-1385.58 to	0.813	0.813

High	-5507.18)			3004.92)			-5930.97)			-16.82)			-35.06)			1765.51)		

NGR, Normal glucose regulation; Pre-DM, Prediabetes mellitus; DM, Diabetes mellitus;

Figure S1. flow chart

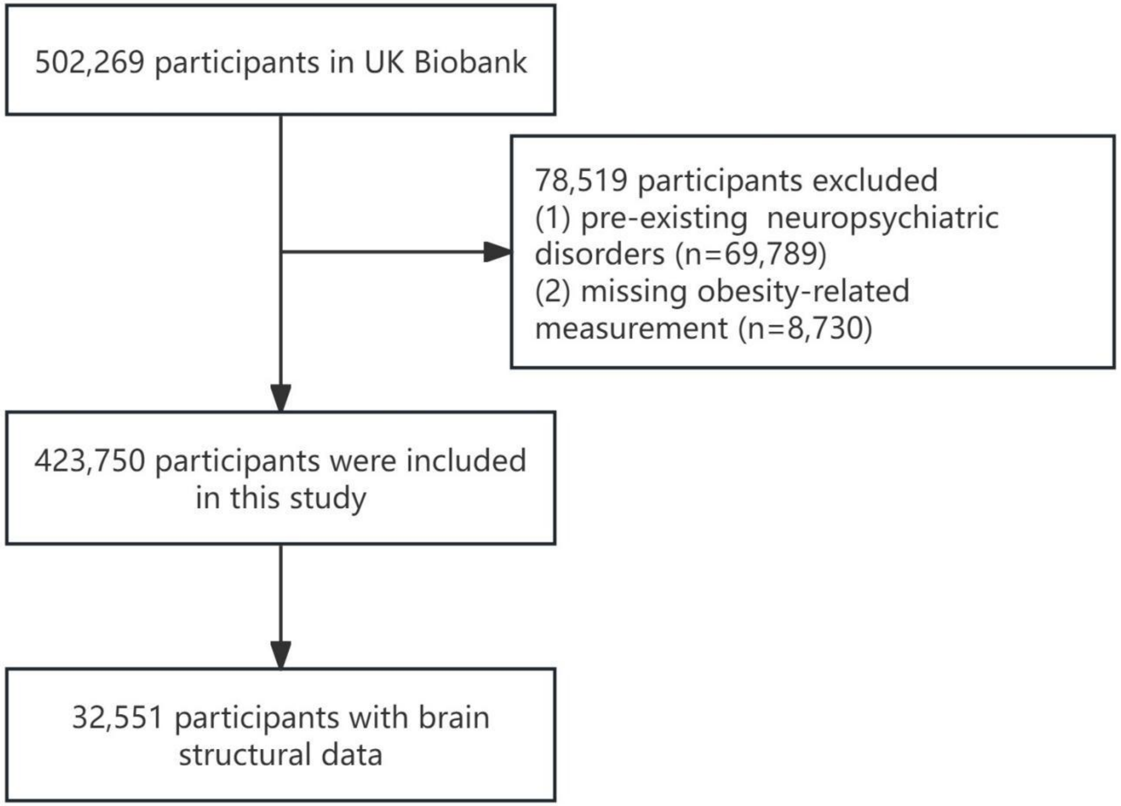

Supplement: Supplementary file 1 [file Table_1.pdf]
